# Genome-Wide Identification of *TaSAUR* Gene Family Members in Hexaploid Wheat and Functional Characterization of *TaSAUR66-5B* in Improving Nitrogen Use Efficiency

**DOI:** 10.3390/ijms23147574

**Published:** 2022-07-08

**Authors:** Weizeng Lv, Xue He, Haojuan Guo, Haibin Lan, Yanqing Jiao, Le Li, Yanhao Lian, Zhiqiang Wang, Zeyu Xin, Yongzhe Ren, Tongbao Lin

**Affiliations:** 1 State Key Laboratory of Wheat and Maize Crop Science, Collaborative Innovation Center of Henan Grain Crops, College of Agronomy, Henan Agricultural University, Zhengzhou 450002, China; lvweizeng@163.com (W.L.); guohaoj1023@163.com (H.G.); lanhaibin1997@163.com (H.L.); jiao673732962@163.com (Y.J.); li_3911@163.com (L.L.); lianyanhao@henau.edu.cn (Y.L.); wzq78@163.com (Z.W.); zeyuxin@yahoo.com (Z.X.); 2 State Key Laboratory of Plant Cell and Chromosome Engineering, Institute of Genetics and Developmental Biology, Innovation Academy for Seed Design, Chinese Academy of Sciences, Beijing 100101, China; hexue@genetics.ac.cn

**Keywords:** *TaSAUR* gene family, *TaSAUR66-5B*, root, nitrogen utilization efficiency

## Abstract

Excessive input of nitrogen fertilizer not only causes a great waste of resources but brings about a series of ecological and environmental problems. Although *Small Auxin Up-regulated* *RNAs* (*SAURs*) participate in diverse biological processes, the function of *SAURs* in the nitrogen starvation response has not been well-studied. Here, we identified 308 *TaSAUR*s in wheat and divided them into 10 subfamilies. The promoter regions of most *TaSAURs* contain hormone responsive elements, and their expression levels change under the treatment of different hormones, such as IAA, MeJA, and ABA. Interestingly, overexpression of one of the *TaSAUR* family members, a nitrogen starvation responsive gene, *TaSAUR66-5B*, can promote the growth of *Arabidopsis* and wheat roots. In addition, overexpression of *TaSAUR66-5B* in *Arabidopsis* up-regulates the expression levels of auxin biosynthesis related genes, suggesting that overexpression *TaSAUR66-5B* may promote root growth by increasing the biosynthesis of auxin. Furthermore, overexpression of *TaSAUR66-5B* in wheat can increase the biomass and grain yields of transgenic plants, as well as the nitrogen concentration and accumulation of both shoots and grains, especially under low nitrogen conditions. This study provides important genomic information of the *TaSAUR* gene family and lays a foundation for elucidating the functions of *TaSAURs* in improving nitrogen utilization efficiency in wheat.

## 1. Introduction

Auxin is the first identified plant hormone, and it influences nearly all aspects of plant growth and development, through the regulation of cell division, expansion, differentiation, and tissue and organ patterning [1]. Auxin regulates hundreds of genes [2] and controls downstream auxin responses via a co-receptor complex that is composed of the *Transport Inhibitor Response 1/Auxin Signaling F-box Proteins* (*TIR1/AFBs*) *and Auxin/Indole Acetic Acid* (*AUX/IAA*) transcriptional repressors [3]. Most early auxin-responsive genes are classified into three families: *AUX/IAAs*, *Gretchen Hagen 3 genes* (*GH3s*), and *Small Auxin Up-regulated* *RNAs* (*SAURs*) [4]. Among these gene families, *SAURs* are the most rapid auxin-responsive genes related to the auxin signaling pathway. Since the first *SAUR* gene was discovered in elongating soybean hypocotyl sections [5], members of this gene family have been identified by genome-wide analyses in diverse plant species, such as *Arabidopsis* [4], rice [6], sorghum [7], tomato [8], potato [8], maize [9], citrus [10], ramie [11], moso bamboo [12], cotton [13], watermelon [14], poplar [15], and apple [16].

Although many *SAUR* genes have been identified in different plant species, only a small portion of them have been functionally characterized. In rice, *OsSAUR39* has been identified as a negative regulator of auxin biosynthesis and transport [17]. In *Arabidopsis*, overexpression of the *AtSAUR19* subfamily genes (*AtSAUR19* to *AtSAUR24*) resulted in increased hypocotyl elongation and leaf size, defective apical hook maintenance, and altered tropic responses [18]. Other *AtSAUR63* (*AtSAUR61* to *AtSAUR68* and *AtSAUR75*) subfamily genes also led to long hypocotyls, petals, and stamen filaments in transgenic *Arabidopsis* [19]. *AtSAUR36* has been reported to play a role in promoting leaf senescence [20]. Many light-induced and/or repressed *SAUR* genes in *Arabidopsis* were also reported to mediate the differential growth of cotyledons and hypocotyls [21]. Gravity- or light-triggered asymmetric auxin distribution induces the asymmetric expression of the *SAUR19* subfamily genes by *Auxin Response Factor 7* (*ARF7*) and *ARF19* in hypocotyls, which leads to bending growth during gravitropic and phototropic responses [22]. A further analysis showed that *AtSAUR19*-stimulated plasma membrane (PM) H^+^-ATPase activity promotes cell expansion by inhibiting the PP2C-D phosphatases [23,24]. The functions of some *SAUR* members have also been identified in wheat. Overexpression of wheat *TaSAUR75* and *TaSAUR78* in *Arabidopsis* enhanced the tolerance of transgenic plants to multiple abiotic stresses [25,26]. These findings show that the *SAUR* genes play important roles in regulating plant growth and development, as well as stress responses.

Wheat (*Triticum aestivum* L.) is one of the three main cereal crops in the world. However, most *TaSAUR* members in hexaploid wheat have not been identified, due to the complexity of its genome. In this study, a genome-wide identification of *TaSAUR* members in wheat, with currently sequenced genomes, was performed to characterize their gene structure, chromosomal locations, and motif compositions. The cis-elements in the promoter regions of all identified *TaSAURs* were analyzed, and the responsive patterns of seven representative *TaSAURs* of different hormones were studied. Overexpression lines of a nitrogen-responsive *SAUR* gene (*TaSAUR66-5B*) in *Arabidopsis* and wheat and RNAi lines in wheat were generated in this study to further probe its roles in low nitrogen adaption.

## 2. Results

### 2.1. Genome-Wide Identification and Characterization of TaSAUR Family Members in Wheat

In this study, 308 *TaSAURs* were identified in wheat (Supplementary Table S1). All predicted wheat *TaSAUR* genes were named from *TaSAUR1-1A* to *TaSAUR172-U*, based on their chromosomal location and genomic homology. The molecular weights of the TaSAUR proteins were 7.64–33.08 kDa, with *TaSAUR107-5D* encoding the highest molecular weight and longest protein (299 aa) and *TaSAUR70-5A* and *TaSAUR84-5B* encoding the lowest molecular weight and shortest protein (71 aa). In addition, the theoretical isoelectric points (PI) ranged from 4.67 (TaSAUR41-5D) to 11.47 (TaSAUR4-2A). The protein subcellular localization prediction showed that 204 of the 308 TaSAUR proteins were located in the mitochondria or chloroplasts. The remaining proteins were predicted to be located in the cytoplasm or nucleus.

### 2.2. Phylogenetic Relationships of SAURs in Five Species

**Figure 1:**
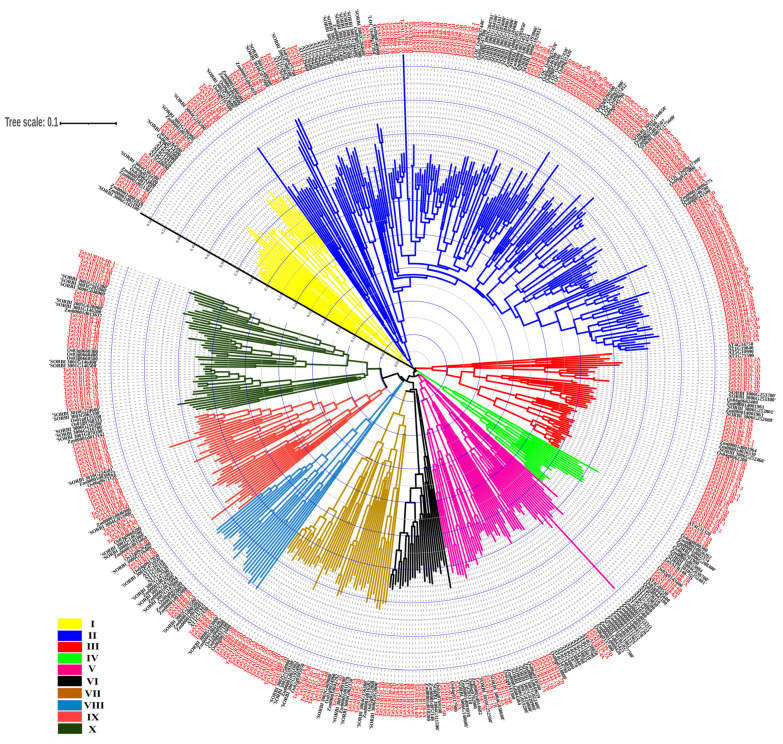
Phylogenetic analysis of SAURs homolog proteins in five different plant species. The phylogenetic tree was generated with MEGA 7.0, using the neighbor-joining (NJ) algorithm. The accession numbers of SAURs proteins in *Oryza sativa*, *Zea mays*, *Arabidopsis thaliana*, *Sorghum bicolor*, and *Triticum aestivum* (red font) are listed in Supplementary Table S2. Ten major clades are distinguished with different colors.

To investigate the evolutionary relationships of *TaSAUR* genes, the SAUR proteins in wheat were compared with those in *Arabidopsis*, rice, maize, and sorghum. A neighbor-joining (NJ) phylogenetic tree was constructed, on the basis of 535 putative nonredundant SAUR protein sequences from five species (Figure 1, Supplementary Table S2). All 535 SAUR proteins were clustered into ten groups, namely group I–group X (Figure 1), which consisted of 38, 185, 51, 19, 59, 25, 44, 24, 41, and 49 members, respectively. Group II is the largest subfamily of TaSAUR. This subfamily contained 121 TaSAUR members (39.3%), of which, 105 were distributed on the fifth homologous group of wheat chromosomes. Group I and VI contained 20 and 17 TaSAUR members, respectively (6.5% and 5.5), of which, 11 and 14 were distributed on the sixth homologous group of wheat chromosomes. Group III contained 37 TaSAUR members (12.0%), of which 35 were distributed on the second homologous group of wheat chromosomes. Group IV is the smallest subfamily. This subfamily contained eight TaSAUR members (2.6%), scattered on the second, sixth, and seventh homologous groups of wheat chromosomes. Group V contained 15 TaSAUR members (4.9%), of which 11 were distributed on the seventh homologous group of wheat chromosomes. Group VII contained 29 TaSAUR members (9.4%), of which 18 were distributed on the fifth homologous group of wheat chromosomes. Group VIII is the smallest subfamily of TaSAURs. This subfamily contained six TaSAUR members (1.9%), and all of them distributed on the fifth homologous group of wheat chromosomes. Group IX and Group X contained 25 and 30 TaSAUR members, respectively (8.1% and 9.7%), of which 15 and 11 members were distributed on the seventh homologous group of wheat chromosomes (Figure 1). Most *TaSAUR* members in the same group, which were unevenly distributed on the fifth, seventh, second, and sixth homologous groups, accounted for 41.9%, 18.2%, 15.9%, and 13.6%, respectively. These results indicate that the *TaSAUR* gene family may have gene expansions on corresponding chromosomes. Additionally, similar events may have occurred in other species. For example, in Group V, most *AtSAUR* genes in *Arabidopsis* were located on chromosome 4 (Figure 1). The overall relationship of the SAURs between wheat and rice was closer than the relationship between wheat and maize, sorghum, or *Arabidopsis.*

### 2.3. Gene Structure and Conserved Motifs of TaSAURs

**Figure 2:**
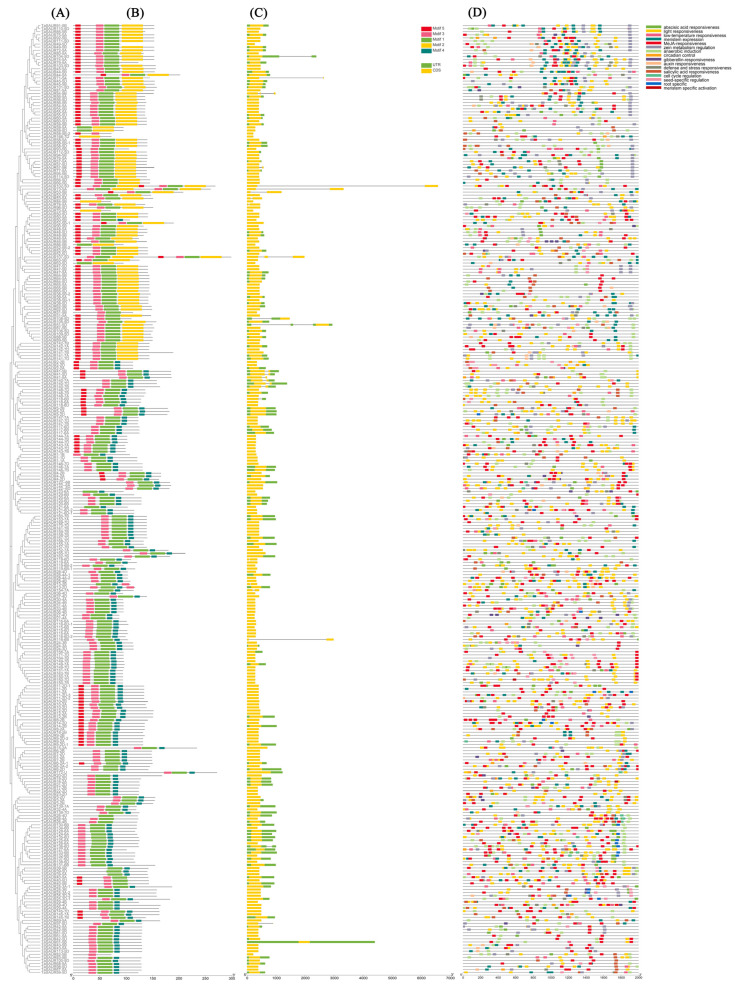
Phylogenetic relationship, motif distribution, gene structure, and cis-element analysis of the TaSAUR proteins and *TaSAUR* genes. (**A**) The phylogenetic tree was constructed using the neighbor-joining method with MEGA 7.0. (**B**) The conserved motifs were predicted using Multiple Em for Motif Elicitation (MEME) (https://meme-suite.org/meme/tools/meme (accessed on 20 January 2022)). The box length indicates the number of amino acids in the motif. (**C**) The structure of *TaSAUR* genes. Yellow boxes represent exons, lines represent introns, and green boxes represent untranslated regions (UTRs). (**D**) The 2 000 bp DNA fragments in the upstream of the coding region of *TaSAUR* genes were selected as putative promoter regions for cis-element analysis using the PlantCARE online tool. Each color indicates one kind of cis-element.

The gene exon–intron structures and conserved motifs were predicted to gain an insight into the variation of the *TaSAUR* genes in wheat. On the basis of the phylogenetic tree (Figure 2A), the structure of different TaSAUR proteins and *TaSAUR* genes were determined, respectively (Figure 2B–C). Motif 1 constituted the conservative SAUR-specific domain and was associated with coding region for auxin-responsive proteins. A total of 98.4%, 33.1%, 95.5%, 62.7%, and 16.2% TaSAUR members contain Motif 1 to 5, respectively (Supplementary Figure S1; Figure 2B). Of all the 308 *TaSAUR* genes identified, 289 genes had no intron (93.8%), while other genes, except for *TaSAUR54-5B* and *TaSAUR107-5D* containing two introns, only had one intron (5.5%) (Figure 2C).

### 2.4. Cis-Elements in the Promoters of TaSAURs

**Figure 3:**
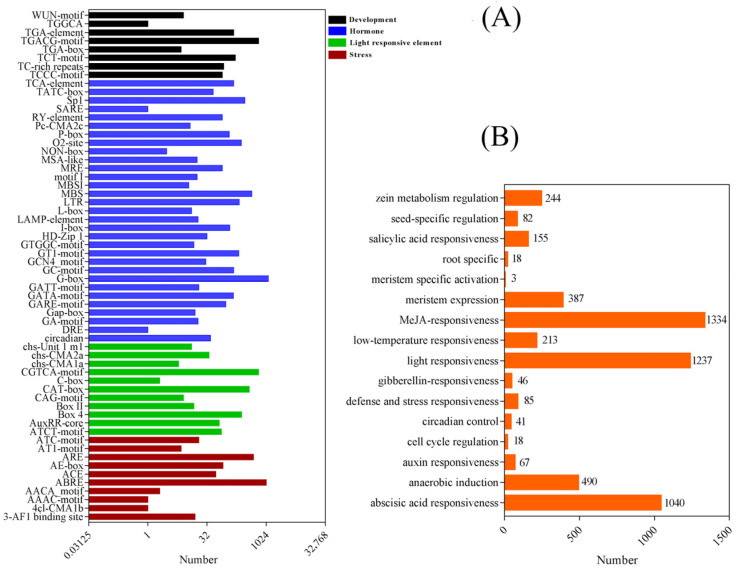
Overview of the main types of cis-elements in the *TaSAUR* gene promoters. (**A**) The number of each type of cis-elements identified in the promoter regions. (**B**) The number of cis-elements identified in the promoter regions in each category.

To further identify cis-elements in the promoter region of *TaSAUR* genes, a 2000 bp DNA fragment in the upstream of the coding region of each putative *TaSAUR* was selected as putative promoter region and analyzed using the PlantCARE tool. As shown in Figure 2D, many kinds of cis-elements, such as light response, development, hormone, and stress-related cis-elements, were identified in the promoters of *TaSAUR* genes. Light-responsive elements, such as G-box, MBS, and TCT, were widely presented in the promoter region of *TaSAUR* genes (Figure 3A). The hormone response elements, including the methyl jasmonate (MeJA)-responsive, abscisic acid-responsive, gibberellin-responsive, salicylic acid-responsive, and auxin-responsive elements, were widely distributed in the promoter region of *TaSAUR* genes. Additionally, many abiotic stress response elements were identified, such as wound, low temperature, anaerobic, and drought stress response-related cis-elements. In addition, some cis-elements related to plant growth and development, such as zein metabolism regulation, flavonoid biosynthesis, seed-specific regulation, meristem expression, endosperm expression, differentiation of the palisade mesophyll cells, meristem specific activation, circadian control, and root-specific and cell cycle regulation, were also found in the promoter regions of *TaSAUR* genes. 

### 2.5. Gene Expression Analysis of TaSAURs under Exogenous Hormones Treatment

**Figure 4:**
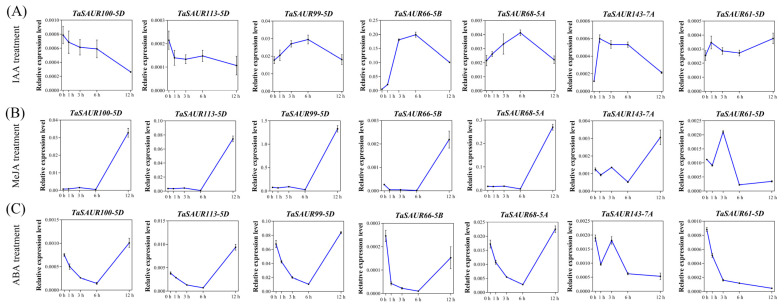
The expression patterns of *TaSAUR* genes under different hormone treatments. Real-time PCR analysis of the expression levels of 7 *TaSAUR* genes were performed in the roots of 2-week-old wheat seedlings under 30 mg·L^−1^ IAA (**A**), 100 μmol·L^−1^ methyl jasmonate (MeJA) (**B**), or 100 μmol·L^−1^ abscisic acid (ABA) (**C**) treatments, respectively. The 2^−^^△Ct^ method was employed to calculate the relative gene expression level, with *β-TaTubulin as* the reference gene. Data are presented as means ± SD of three independent biological replicates.

The promoter regions of *TaSAURs* contain numerous hormone response elements. We used real-time quantitative PCR to analyze the expression patterns of seven representative *TaSAUR*s with high expression level in wheat roots in response to different hormones. Five of them (*TaSAUR100-5D*, *TaSAUR113-5D*, *TaSAUR99-5D*, *TaSAUR61-5D*, and *TaSAUR68-5A*) were expressed specifically in roots, and two (*TaSAUR66-5B* and *TaSAUR143-7A*) were expressed in roots, stems, and leaves. Results showed that the expression levels of *TaSAUR100-5D* and *TaSAUR113-5D* down-regulated in response to IAA treatment, while *TaSAUR99-5D*, *TaSAUR61-5D*, *TaSAUR68-5A*, *TaSAUR66-5B*, and *TaSAUR143-7A* were all up-regulated (Figure 4A). These results verified that *TaSAUR*s were early responsive genes to auxin, but the response patterns of different members have significant differences, with some up-regulated and some down-regulated.

*TaSAUR100-5D*, *TaSAUR113-5D*, *TaSAUR99-5D*, *TaSAUR66-5B*, *TaSAUR68-5A*, and *TaSAUR143-7A* were up-regulated after 6 h MeJA application and reached the maximum expression levels at 12 h. While the expression of *TaSAUR61-5D* was up-regulated at first, it then down-regulated, reaching the peak at 3 h and lowest at 6 h, and then increased gently (Figure 4B). For ABA treatment, the expression of *TaSAUR100-5D*, *TaSAUR113-5D*, *TaSAUR99-5D*, *TaSAUR66-5B*, and *TaSAUR68-5A* were down-regulated at 0–6 h and then up-regulated continuously. *TaSAUR143-7A* and *TaSAUR61-5D* were down-regulated and reached the lowest point at 12 h (Figure 4C). The expression of *TaSAUR100-5D*, *TaSAUR113-5D*, *TaSAUR99-5D*, *TaSAUR66-5B*, and *TaSAUR68-5A* were up-regulated by exogenous ABA or MeJA, but there were differences in expression levels. *TaSAUR61-5D* was down-regulated by ABA or MeJA at 12 h, which may play a negative role in stress responses. The expression pattern of *TaSAUR143-7A* changed dynamically under the two conditions, but the relative expression level was opposite at 12 h; that is, it was down-regulated by ABA and up-regulated by MeJA, suggesting that MeJA may be the main signal pathway for the positive regulation of the *TaSAUR143-7A* gene under adverse conditions.

### 2.6. Functional Analysis of TaSAUR66-5B

**Figure 5:**
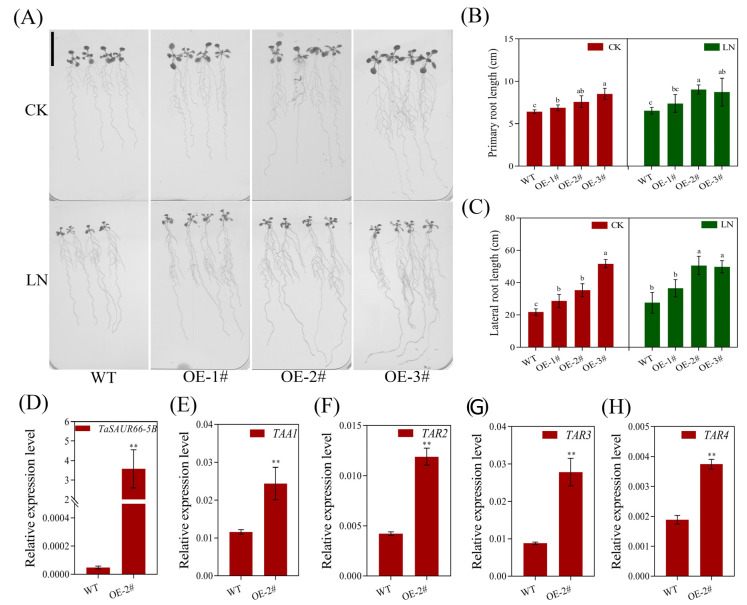
Overexpression of *TaSAUR66-5B* in *Arabidopsis thaliana* up-regulates key genes in auxin biosynthesis and promotes root growth. (**A**) Root phenotypes of *Arabidopsis* transgenic lines under normal nitrogen (CK) and low nitrogen (LN) conditions. Bar = 2 cm. (**B**) The primary root length of *Arabidopsis* wild-type (WT) and transgenic lines. (**C**) The lateral root length of WT and transgenic lines. (**D**–**H**) The relative expression levels of key genes in auxin biosynthesis *TAA1, TAR2, TAR3*, and *TAR4* in WT and transgenic line. The relative expression levels were normalized to the expression of *AtActin* by 2^−^^△^^Ct^. ** indicates significant difference (*p* < 0.01). Different lower case letters above columns indicate statistical differences at *p* < 0.05 level.

**Figure 6:**
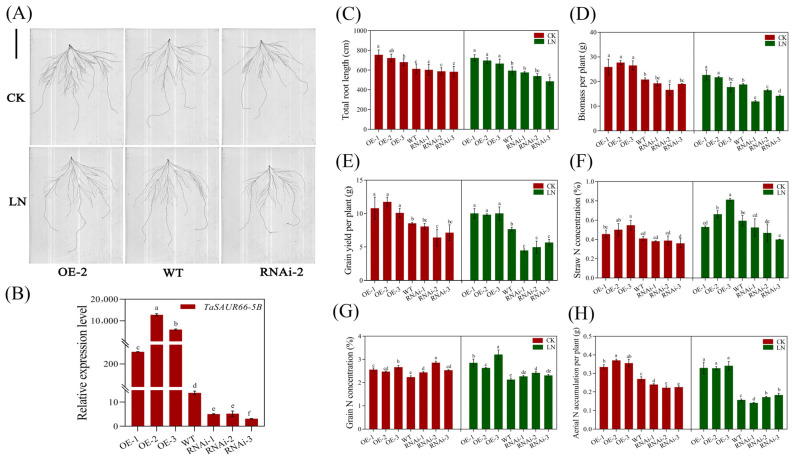
Overexpression of *TaSAUR66-5B* in wheat promotes root growth and improves nitrogen use efficiency (NUE). (**A**) The root morphology of representative plants of WT and transgenic line under normal nitrogen (CK) and low nitrogen (LN) conditions. Bar = 10 cm. (**B**) Relative expression levels of *TaSAUR66-5B* gene in different wheat transgenic lines. The relative expression level of *TaSAUR66-5B* was normalized to the expression of *β-TaTubulin*, 2^−^^△Ct^. (**C**) The total root length of each wheat transgenic line under CK and LN conditions. (**D**) The biomass per plant, (**E**) grain yield per plant, (**F**) straw nitrogen concentration, (**G**) grain nitrogen concentration, and (**H**) aerial nitrogen accumulation of transgenic lines and WT under CK and LN conditions, respectively. The data are expressed as the mean ± SD of three biological replicates. Different lower case letters above columns indicate statistical differences at *p* < 0.05 level.

Since the response of *TaSAUR66-5B* to auxin is the most significant one among the seven investigated *TaSAUR* genes, and it can respond to low nitrogen stress based on one of our microarray analyses, we, therefore, performed further analysis on it. Firstly, we examined the expression levels of *TaSAUR66-5B* in the roots of a pair of wheat isogenic lines, i.e., 178A and 178B [27]. We found that the expression of *TaSAUR66-5B* was significantly higher in the long-root line 178B, compared to that in the short-root line 178A, and significantly up-regulated in both 178A and 178B under low nitrogen conditions. The expression levels increased by 60.1 times in 178B under low nitrogen, while they increased by 3.88 times in 178A (Supplementary Figure S2). These results indicated that *TaSAUR66-5B* might be involved in the regulation of wheat root growth and low nitrogen adaption. Then, we generated overexpression lines of *TaSAUR66-5B* in *Arabidopsis* and wheat and RNAi lines in wheat to further probe its biological function (Figure 5A and Figure 6A). Phenotypic analysis of homozygous T3 *Arabidopsis* transgenic lines showed that the primary and lateral roots of transgenic plants were significantly longer than those of WT under both low nitrogen (LN) and normal nitrogen (CK) conditions (Figure 5A–C), thus indicating that overexpression of *TaSAUR66-5B* in *Arabidopsis* significantly promoted the root growth of transgenic plants. We examined the expression levels of four key genes (*TAA1*, *TAR2*, *TAR3*, and *TAR4*) involved in auxin biosynthesis. Results showed that the relative expression levels of the four examined genes were all significantly up-regulated in the *TaSAUR66-5B* overexpressed lines (Figure 5D–H). Taken together, *TaSAUR66-5B* may enhance root growth by promoting auxin biosynthesis.

Similarly, overexpression of *TaSAUR66-5B* in wheat also significantly promoted the root growth of transgenic plants under both LN and CK conditions (Figure 6A–C). However, the effects of underexpression of *TaSAUR66-5B* on root growth in RNAi plants is not significant (Figure 6C). The most likely reason is that there are a large number of *TaSAUR* gene family members and the presence of function redundancy in hexaploid wheat. In the field trial, the biomass and grain yield of *TaSAUR66-5B* overexpression lines were significantly higher than those of WT under both LN and CK conditions (Figure 6D,E). The biomass increased by 24.62–33.32% under CK and 15.50–20.28% under LN conditions. The grain yield increased by 18.17–37.59% under CK and 27.96–30.76% under LN conditions. The *TaSAUR66-5B* underexpression lines (RNAi lines) had significant lower biomass and grain yield than those of WT under LN conditions but no significant lower biomass and grain yield under CK. To investigate whether *TaSAUR66-5B* can improve the nitrogen use efficiency (NUE) in wheat, we measured the nitrogen concentration of the straw and grains of *TaSAUR66-5B* transgenic lines and calculated the nitrogen accumulation based on straw biomass and grain yield. Results showed that the nitrogen concentration and nitrogen accumulation in the straw and grains of almost all overexpression lines were significantly higher than those of WT, especially under LN conditions (Figure 6F–H). These results suggest that overexpression of *TaSAUR66-5B* can improve wheat growth performance, yield, and NUE, especially under LN conditions. Similar to the effects on root growth, underexpression of *TaSAUR66-5B* on nitrogen uptake in most RNAi plants is not significant, which, once again, indicates the possible functional redundancy among the *TaSAUR* gene family members.

## 3. Discussion

Auxin, an important plant hormone, forms a complex signaling network with other plant hormones [28,29]. The *SAUR* gene family is one of three early auxin-responsive gene families; however, the function of most *SAUR* genes remains unknown. One possible reason is that there are numerous members in *SAUR* gene family, especially in hexaploid wheat. The complex genome of hexaploid wheat adds a barrier to study the function of *TaSAUR*s. With the completion of wheat genome sequence, the genome-wide identification of *TaSAUR* gene family members becomes feasible. Here, we identified *TaSAUR* family members and predicted the characteristics, chromosomal locations, subcellular localization, and cis-elements of each member. We totally identified 308 *TaSAUR* members in hexaploid wheat. Motif analysis showed that 287 of the 308 TaSAURs possess motif 1 andmotif 3 (Figure 2B), thus indicating that the domains are strongly conserved in TaSAUR family. The majority of *TaSAUR* genes (93.8%) have no intron. This is consistent with the results in other species. For example, none of the *SAURs* in rice have any intron, whereas only one *SAUR* gene in *Arabidopsis* harbors an intron [6]. Different SAUR members may localize in different subcellular organelles, such as the nucleus [30], cytoplasm [17], or PM [18], and play different biological roles. In the present study, more than 66% TaSAURs were localized in the mitochondria or chloroplasts. This feature has also been found in other species. In fact, more than half of the ZmSAURs proteins in maize and PtSAURs in poplar are predicted to be localized in mitochondria or chloroplasts [9,15]. Mockevi et al. (2006) [31] found that some IAA auxin-binding protein complexes may be localized in the mitochondria and chloroplast, indicating that auxin may be involved in the first energy transfer processes that occur on the membrane of both organelles. As an early auxin-responsive gene family, TaSAUR proteins may also be involved in this process. In *Arabidopsis*, *SAUR19* overexpression and *SAUR19/23/24 artificial microRNA* (*amiRNA*) knockdown seedlings conferred an increased and decreased basipetal indole-3-acetic acid (IAA) transport, respectively, in hypocotyls [18]. Similar findings have been obtained with *SAUR41* and *SAUR63* [19,32]. *Arabidopsis SAUR15* and *SAUR70* have been shown to bind CaM or CaM-related proteins [33]. The above genes, with similar function, were clustered into the same branch in the evolutionary tree (Figure 1), which indicated the feasibility of analyzing the conservative function of genes by the evolutionary tree. 

Many light-, hormone-, and stress-responsive elements were identified in the promoter regions of *TaSAURs* (Figure 3). Some elements are closely related to the regulation of plant growth and development. For example, the G-box element has been reported to be involved in the regulation of chlorophyll II biosynthesis in *Arabidopsis* [34]. Thus, *TaSAURs* may be involved in the regulation of plant growth and development through these cis-elements. Studies has shown that the core component of BR signaling, BZR1/BES1, can bind to the promoters *of* *SAUR15,* *SAUR36*, and *SAUR59* in *Arabidopsis* [35,36,37,38,39]. Bai et al. (2012) found that 27 *SAURs* genes were up-regulated, following a 12 h GA treatment [40]. Many *SAURs* genes exhibit reduced expression in response to ABA treatment, as well as drought and osmotic stresses [41]. Since the promoter regions of *TaSAURs* also have a large number of cis-acting elements in response to auxin, ABA and MeJA (Figure 3), we, therefore, analyzed the expression pattern of seven selected *TaSAURs* under different hormone treatments and found that most of them respond quickly to IAA, ABA, and MeJA (Figure 4). Both ABA and MeJA have been shown to enhance the ability of plants to cope with stress. Previous studies have shown that the JA biosynthesis pathway may regulate root structure by regulating JA level, thus improving nutrient utilization efficiency and tolerance to N, P, and K deficiency in rice [42]. Under the condition of heavy metal cadmium (Cd), exogenous ABA application significantly increased nitrate reductase (NR) activity in roots by 82.8%, GS activity in shoots by 32.1%, and NUE by 17.2% [43].

The regulation of nitrogen utilization by auxin may be related to *NRT1.1*, which not only encodes protein as a nitrate transporter but also promotes auxin polar transport and regulates lateral roots development in *Arabidopsis thaliana* [44,45,46,47]. *DULL NITROGEN RESPONSE1* (*DNR1*) is another crucial gene negatively regulating NUE by auxin. Rice plants without the *DNR1* gene loci showed increased auxin synthesis, NUE, and yield [48]. In this study, we found that overexpression of a nitrogen-responsive *SAUR* gene (*TaSAUR66-5B*) promoted root growth, and the key genes for auxin biosynthesis were significantly upregulated in transgenic plants (Figure 5). As a matter of fact, some members of *SAURs* in *Arabidopsis*, *SAUR76*, *SAUR15*, and *SAUR41* can positively regulate roots growth, mainly associated with activation of H^+^-ATPase and auxin biosynthesis and transport [32,49,50]. It has been reported that *AtSAUR41* initiates and regulates cell expansion and root meristem establishment [32,51]. During lateral root development, *SAUR41* is specifically expressed in the potential stem cell niches of lateral root primordia and enlarged endoderm cells around the primordia. *OsSAUR39* has been reported to negatively regulate root development, reduce lateral root number, and stem and root length in rice [17]. Overexpression *OsSAUR45* showed pleiotic developmental defects, including reduced plant height and primary root length, secondary roots, narrower leaves, and lower seed setting rate [52]. *OsSAUR39* and *OsSAUR45* are considered to be negative regulators of auxin synthesis and transport. However, although TaSAUR66-5B and OsSAUR39 are on the same branch of the evolutionary tree (Figure 1), their biological functions are quite different. *TaSAUR66-5B* exhibits a positive regulation of auxin biosynthesis and root growth. Overexpression of *TaSAUR66-5B* promoted auxin biosynthesis and root growth (Figure 5 and Figure 6A–C). These results means that *TaSAUR* gene family has complex functional differentiation. Even different *TaSAUR* members in the same group may also have different functions. The auxin biosynthetic gene *TAR2* has also been reported to regulate root system architecture under LN conditions. Studies have found that LN treatment can induce *TAR2* and promote the auxin accumulation. Excessive exogenous IAA treatment can down-regulate *TAR2* to balance the auxin concentration in lateral roots. Auxin concentration increased with the decrease of nitrogen level, and the formation of lateral roots could be induced by the auxin biosynthesis and accumulation under LN stress [53,54]. In this study, the expression of *TaSAUR66-5B* was rapidly up-regulated by IAA treatment (Figure 4A), while overexpression of *TaSAUR66-5B* also promoted the biosynthesis of auxin (Figure 5E–H). These results mean that, as early response genes of auxin, *SAURs*, in turn, affect auxin biosynthesis. Similarly, a previous study in rice showed that *OsSAUR39* reached the highest expression level at 8 h under NAA induction, while overexpression of *OsSAUR39* resulted in decreased auxin level and transport [17]. Those evidence suggest that such feedback regulation is common in *TaSAURs* gene family. As plant roots are the main organs of nutrient uptake from soil, the genetic improvement of root traits is crucial to improving crop nutrient use efficiency [55,56,57]. In this study, we observed that overexpression of *TaSAUR66-5B* not only increases the nitrogen concentration and nitrogen accumulation of both shoots and grains but also the biomass and grain yield (Figure 6). Therefore, *TaSAUR66-5B* could be a target gene in future genetic improvement of wheat root traits and NUE.

## 4. Materials and Methods

### 4.1. Plant Materials

The wheat variety Kenong 199 (KN199) was used in this study to isolate the sequence and check the expression level of *TaSAUR66-5B* and generate transgenic lines. The Columbia (Col-0) was used to generate *Arabidopsis thaliana* transgenic lines. *TaSAUR66-5B* was cloned and overexpressed in *Arabidopsis* following the procedures for the *TAR2* in a previous study [53].

KN199 was used in this study for genetic transformation. To generate overexpression lines, the cDNA of *TaSAUR66-5B* were inserted into the *pUbi-163* vector, resulting in *pUbi::TaSAUR66-5B* constructs. To generate RNAi lines, the conserved sequence of *TaSAUR66-5B* was inserted into a *pUbi-RNAi* vector, resulting in *pUbi::TaSAUR66-5B-RNAi* constructs. The above constructs were then transformed into immature embryos of wheat variety KN199, respectively, as described previously [58]. The primers used for vector construction and expression level analysis are listed in Supplementary Table S3. The wild-type KN199 and T3 transgenic wheat lines were used as materials in the hydroponic culture and field experiment.

### 4.2. Identification and Classification the TaSAUR Members in Wheat

The SAUR amino acid sequences of *Arabidopsis*, rice, sorghum, and maize were downloaded from the Ensemble Plants databases (http://plants.ensembl.org/index.html (accessed on 20 January 2022)), respectively. Candidate sequences were inspected with Hidden Markov Model (HMM) implemented with default parameters in HMMER v3.3.2 (https://www.ebi.ac.uk/Tools/hmmer/search/hmmscan (accessed on 20 January 2022)) to confirm the presence of the conserved SAUR domain (PF02519). All TaSAURs sequence information was obtained according to Pfam ID in the wheat database (IWGSC RefSeq v1.1). The Pfam database (http://www.sanger.ac.uk/Software/Pfam/search.shtml (accessed on 20 January 2022)) was used to confirm each predicted TaSAUR protein containing a conserved SAUR-specific domain.

### 4.3. Characterization of Predicted TaSAUR Proteins

The ProtParam tool (https://web.expasy.org/protparam/ (accessed on 22 January 2022)) was used to analyze the physicochemical parameters (i.e., length, molecular weight, and isoelectric point) of SAUR proteins. Subcellular localization prediction was conducted using the CELLO v2.5 server (http://cello.life.nctu.edu.tw/ (accessed on 22 January 2022)).

### 4.4. Analysis of Conserved TaSAUR Motifs and Gene Structures

The gene structure, intron/exon distribution and intron/exon boundary of *TaSAURs* were annotated using the GFF3 file containing wheat genome information. The Multiple Em for Motif Elicitation and MAST Primer Search (http://meme-suite.org/tools/meme (accessed on 22 January 2022)) program were used to analyze the protein sequences of wheat. The following parameter settings were used: each sequence contain at most one occurrence of each motif, the number of different motifs was 5, and motif length ranged from 6 to 50 amino acids. TBtools software (https://github.com/CJ-Chen/TBtools (accessed on 22 January 2022)) was used for graphical visualization [59].

### 4.5. Phylogenetic Analysis

Multiple sequence alignments for SAUR full-length protein sequences of *Arabidopsis*, rice, maize, sorghum, and wheat were performed using the Multiple sequence alignment version 3 (MUSCLE v3) (http://www.drive5.com/muscle/ (accessed on 22 January 2022)). On the basis of the neighbor-joining method, a phylogenetic tree was constructed for SAUR proteins by using the software MEGA 7.0. The bootstrap test was carried out using 1000 replicates to construct the reliability of the tree. A midpoint rooted base tree was produced using the Interactive Tree of Life (iToL, version 6.4.2, https://itol.embl.de (accessed on 22 January 2022)).

### 4.6. Analysis of Cis-Elements

To survey cis-regulatory elements in the promoter regions of *TaSAURs*, the 2000 bp DNA fragments in the upstream of the coding region of *TaSAUR* genes were selected as putative promoter regions for cis-element scanning. The sequences of *TaSAURs* promoter regions were obtained from IWGSC RefSeq v1.1 database (https://plants.ensembl.org/Triticum_aestivum/Info/Index (accessed on 20 October 2021)). The PlantCARE program (https://bioinformatics.psb.ugent.be/webtools/plantcare/html/ (accessed on 20 October 2021)) was used to identify cis-regulatory elements in the putative promoter regions.

### 4.7. Growth Conditions and Treatments in the Hydroponic Culture

Hydroponic culture was used to evaluate wheat growth and gene expression of the wild-type KN199 and transgenic lines at seedling stage. The growth conditions and N treatments used in this study were as previously described [60,61]. After 14 d, the roots were harvested for root morphological analysis with six biological replicates. The root morphological parameters were analyzed using WinRHIZO software (Regent Instruments Canada), as described by Ren et al. (2012) [61]. In the hormones treatment trial, KN199 seedlings were grown under normal N (CK) conditions for 14 days; then, the leaves were sprayed with IAA, ABA, and MeJA (Aladdin, Shanghai, China), respectively. For IAA (30 mg·L^−1^), ABA, and MeJA (100 μmol·L^−1^) treatment, the roots of four plants were collected at 0, 1, 3, 6, and 12 h after hormone treatment, respectively, and stored at −80 °C for RNA extraction and, subsequently, gene expression analysis.

The *Arabidopsis* growth conditions and N treatment methods were as previously described [53]. After being grown vertically for 5 d, the roots were scanned using WinRHIZO software. The scanned images were then used to measure root length using ImageJ software.

### 4.8. Field Experiment

The field experiment was conducted in the 2019–2020 wheat growing season at the experimental station of the Institute for Cereal and Oil Crops, Hebei Academy of Agriculture and Forestry Sciences, Hebei Province, China. The experiment consisted of two N conditions, i.e., normal nitrogen (CK) and low nitrogen (LN) conditions. In the CK treatment, 18 g m^−2^ N, in the form of diammonium phosphate (24 g m^−2^) and urea (30 g m^−2^), were applied. In the LN treatment, only diammonium phosphate (24 g m^−2^) was applied. Three biological replicates of each treatment were conducted. For each genotype in each replicate, 40 seeds were sown in one 2 m-long row, and the rows were spaced 23 cm apart. At maturity stage, 15 representative plants were harvested, in order to investigate the biomass and grain yield in each replicate. N concentration in the straw and grain were measured using a semiautomated Kjeldahl method (Kjeltec Auto 1030 Analyzer, FOSS, Denmark).

### 4.9. Real-Time Quantitative PCR and Data Analysis

Total RNA was isolated using *M5* SuperPuretotal RNA extraction reagent (SuperTRIgent) (*Mei5bio*, Beijing, China). First-strand cDNAs were produced from the RNA template by reverse transcription, using the *FastKing* RT Kit (With gDNase) (*TianGen*, Beijing, China). Real-time quantitative PCR was performed on an ABI Step-one thermal cycler, using the real-time PCR Master Mix (*Mei5bio*, Beijing, China).

Relative expression levels for the target genes were normalized using the housekeeping genes *β-TaTubulin* (U76558, in wheat) and *AtActin2* (*NM_112764*, *AT3G18780*, in *Arabidopsis thaliana*), respectively. The primers used are listed in Supplementary Table S3.

### 4.10. Statistical Analysis of Data

One-way ANOVA was performed using SPSS21 for Windows.

## 5. Conclusions

We totally identify 308 *TaSAUR* gene family members in wheat genome and divide them into 10 subfamilies. Most *SAURs* promoters contain hormone response elements and can respond to different hormones. Overexpression of a nitrogen starvation responsive *TaSAUR* gene, *TaSAUR66-5B*, can promote root growth, possibly by increasing auxin biosynthesis. Moreover, overexpression of *TaSAUR66-5B* in wheat can increase the nitrogen concentration, nitrogen accumulation, and biomass and grain yield of transgenic plants, especially under LN conditions. Our results provide comprehensive information for the wheat *TaSAUR* gene family and a genetic resource for improving nitrogen utilization efficiency in wheat.

## Data Availability

All data are reported in this manuscript.

## Supplementary Materials

The following supporting information can be downloaded at: https://www.mdpi.com/article/10.3390/ijms23147574/s1.
