# Supplementary figures and images for: Genome-Wide Identification of TaSAUR Gene Family Members in Hexaploid Wheat and Functional Characterization of TaSAUR66-5B in Improving Nitrogen Use Efficiency
Source: Int J Mol Sci. 2022 Jul 8;23(14):7574. doi: 10.3390/ijms23147574 (PMC9319360; doi:10.3390/ijms23147574)

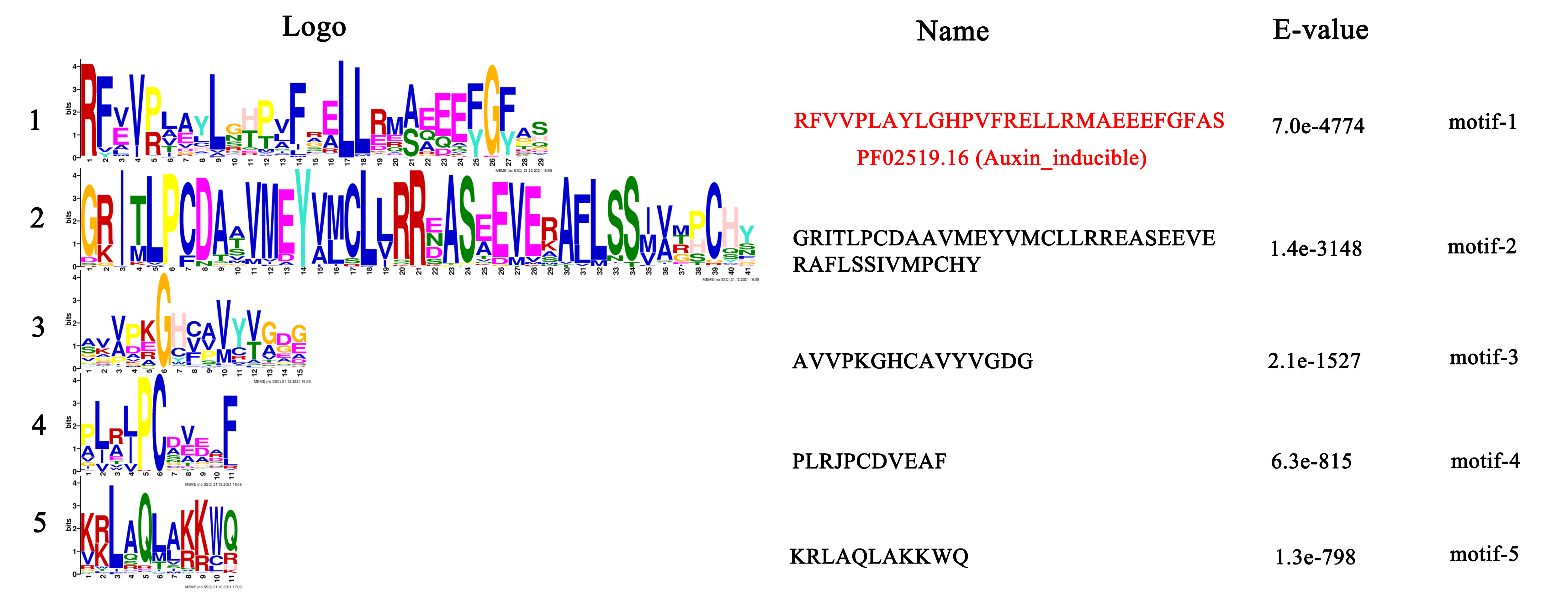

Supplement: Supplementary file 1 [file ijms-23-07574-s001.zip › Figure S1 Discovered motifs in TaSAURs.tif]

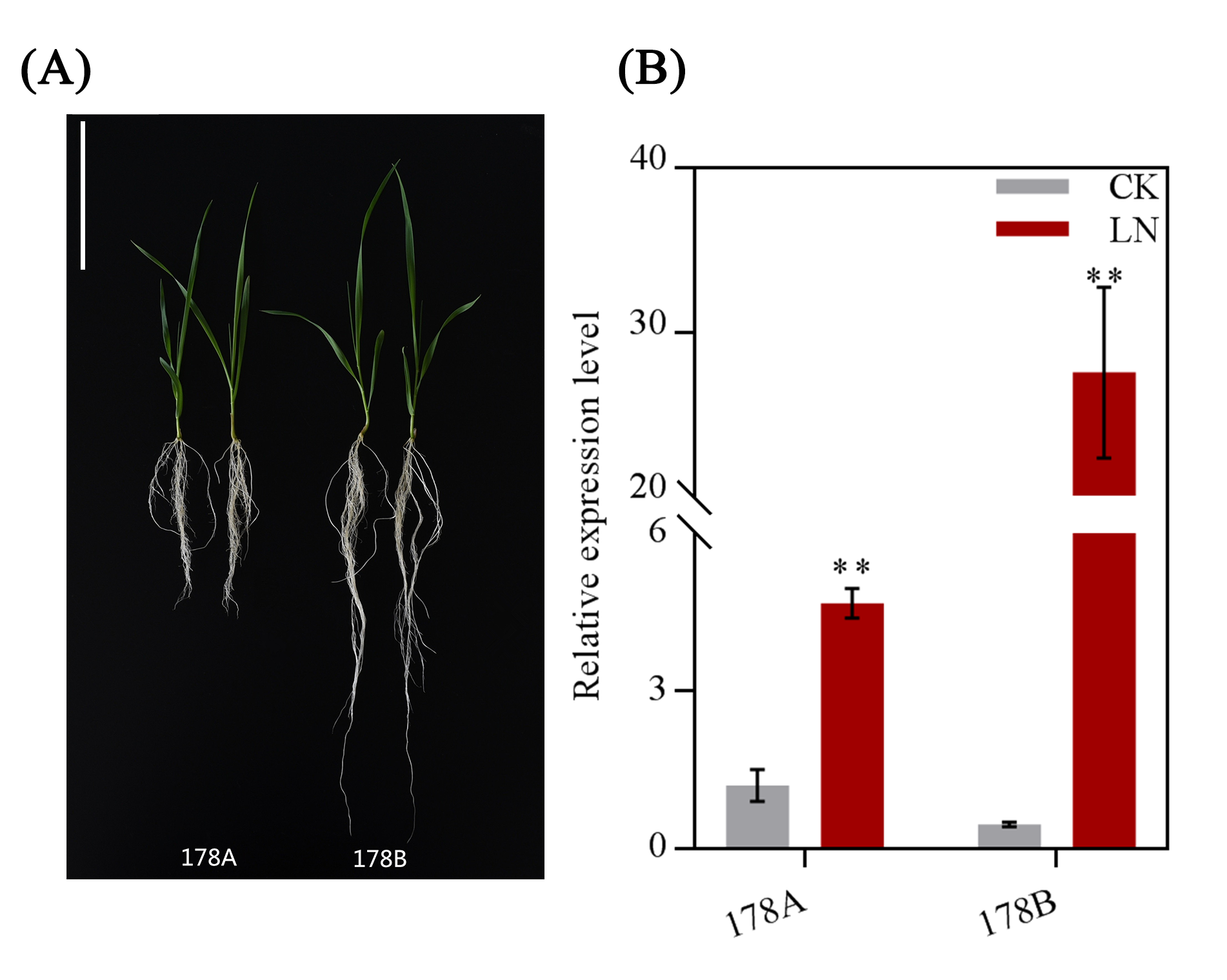

Supplement: Supplementary file 1 [file ijms-23-07574-s001.zip › Figure S2 Root morphology and relative expression levels of the TaSAUR66-5B gene in the roots of 178A and 178B isogenic lines.tif]
